# Negative effect of methyl bromide fumigation work on the central nervous system

**DOI:** 10.1371/journal.pone.0236694

**Published:** 2020-08-03

**Authors:** Min-Goo Park, Jungmi Choi, Young-Seoub Hong, Chung Gyoo Park, Byoung-Gwon Kim, Se-Young Lee, Hyoun-Ju Lim, Hyoung-ho Mo, Eunjo Lim, Wonseok Cha

**Affiliations:** 1 Department of Plant Quarantine, Division of Pest Control, Animal and Plant Quarantine Agency (APQA), Gimcheon-si, Gyeongsangbuk-do, Republic of Korea; 2 Institute of Life Science (BK21^+^ Program), Gyeongsang National University, Jinju, Gyeongnam, Republic of Korea; 3 Human Anti-Aging Standards Research Institute, Uiryeong-gun, Gyeongsangnam-do, Republic of Korea; 4 Department of Preventive Medicine, College of Medicine, Dong-A University, Busan, Republic of Korea; 5 Heavy Metal Exposure Environmental Health Center, Dong-A University, Busan, Republic of Korea; VIT University, INDIA

## Abstract

Methyl bromide (MB) is a fumigant that has been widely used for killing pests on plants in trade, soils, and structures worldwide due to its excellent permeability and insecticidal effect; however, MB should be replaced because it is an ozone-depleting substance. It is well-known that MB is highly toxic and hazardous to workers, but the effects of exposure in asymptomatic workers have not been explored. The purpose of this study is to investigate the impact of MB fumigation on the health of fumigators at a sensitive level. The electroencephalogram (EEG) and urinary bromide ion levels of 44 fumigators (the study group) and 20 inspectors (the control) were measured before and after fumigation work from February to August 2019 in Busan, Korea. The mean post-work concentration of bromide ion (18.311 μg/mg CRE) in the fumigators was significantly increased from the pre-work level (7.390 μg/mg CRE) (*P*<0.001). The fumigator post-work median frequencies (MDF) and alpha-to-theta ratios (ATR) of EEG index were significantly decreased compared to the pre-work values (*P*<0.05 for all indices). In contrast, there were no significant differences in inspector EEG indices and urinary bromide ion. The urinary bromide ion levels in all the subjects were negatively correlated with MDF (*P* = 0.032). In conclusion, fumigators’ EEG indices and urinary bromide ion suggested that occupational exposure to MB negatively affected the health of workers, although the workers were asymptomatic.

## Introduction

Methyl bromide (MB) has been applied worldwide for quarantine treatment for plants in trade and to control structural pests in buildings since its efficacy was first reported in 1930 [1–3]. It has also been used widely as a soil fumigant for killing pathogens, nematodes and weeds.

In 1992, the United Nations Environment Programme (UNEP) listed MB as an ozone-depleting substance under the Montreal Protocol and stated that developed countries should stop using MB by 2005, while developing countries should stop use by 2015; however, the parties anticipated that no alternatives to MB would arise during that timeframe that gave the same level of efficacy for the diverse range of treatments for which MB is used, and the use of MB for quarantine, pre-shipment (QPS) and Critical Use Exemption (CUE) purposes—for agricultural use where there are no technical or financial alternatives—were excluded from the phase-out programme established in the protocol [4,5]. The consumption of MB was reduced considerably from 31,793 tons in 2005 to 12,709 tons in 2013. Nevertheless, the reduction in MB use for QPS purposes is not expected at this time because there has been only a slight change from 10,825 in 2005 to 9,827 tons in 2013 for QPS purposes, while the amount of MB used in 2013 for soil fumigations was 2,882 tons, approximately 86% less than the 20,968 tons used in 2005 [6].

In addition to being an ozone depleting substance, MB is a highly toxic pesticide that causes acute or chronic toxicity to fumigators and related workers [7–14]; a worker at a warehouse that stores fumigated plants showed an unstable gait, vertigo and paraesthesia of both feet due to MB poisoning; fumigators presented polyneuropathy, optic neuropathy, and cerebellar signs with high MB concentrations in the blood and urine; two experienced fumigation workers entered a building with high MB concentrations, rapidly felt unwell and complained of nausea and shortness of breath; and cases of MB toxicity were reported in ships on which containers were fumigated as well as in warehouses.

The main exposure route of MB is respiratory. MB inhaled through respiration is absorbed into the blood through the lungs. The absorbed MB may be directly toxic to cells via its ability to bind lipids and proteins [15]. In addition, MB has been shown to deplete glutathione in several tissues, such as the liver, kidneys, lungs, and brain [15,16]. It is also known that MB absorbed in the human body causes acute lung injury, neurological disorder, optic neuritis, cytotoxicity, genetic toxicity and various toxic activities related to metabolism [7,8,11,17]. Neurotoxicity in particular appears to be the most sensitive effect of acute exposure in animals [18].

Biomarkers to determine whether workers have been exposed to MB include bromide ions in the blood, serum or urine [16,19,20]. Even if the bromide ion in the fumigator’s urine is detected at levels higher than that of ordinary people, the symptoms of poisoning do not always appear [19,21]. It is unclear why asymptomatic fumigators do not show health effects even with high bromide ion concentrations in their urine. This may be because symptom onset differs between individuals [21]. A biomarker that can sensitively assess workers’ health is needed to determine whether fumigation work affects the health of workers without symptoms.

Neuroscience researchers have long used electroencephalogram (EEG) to evaluate brain function in clinical practice. Median frequency (MDF) and alpha-to-theta ratio (ATR) in resting state EEG could predict future development of Alzheimer’s disease (AD) in mild cognitive impairment (MCI), showing a slowed intrinsic EEG oscillation [22–24]. A slowed intrinsic EEG oscillation was also related to performance decline on psychometric tests, such as the Mini Mental State Examination (MMSE), the Cambridge Cognition Examination (CAMCOG), the Wechsler Memory Scale (WMS), the Global Deterioration Scale (GDS), and the Alzheimer’s Disease Assessment Scale (ADAS) in many studies [25–33]. Recently, thanks to the development of IT technology, a convenient and high quality low-channel wireless EEG measurement system has been widely used [34,35]. The authors of this study have shown that two-channels EEG systems, measured at prefrontal region, show reliable data quality and significant clinical results [36–38]. With two-channels EEG system, the authors have also shown that MDF and ATR are correlated negatively with the MMSE scores of elderly people (total = 496, males = 163, females = 331) [36]

Correlations between EEG changes and neuroimaging findings have been reported in many studies as well. Global alpha showed a relationship to the volumetric changes of sub-cortical white, hippocampus, thalamus and basal ganglia and cortical grey matter [39–42]. In an earlier study, EEG markers could predict dementia severity with similar power as PET markers [43]. Pathologic changes in AD are generally assumed to start several years before initial symptoms of cognitive decline appear [23]. These findings indicate that EEG markers might be useful and sensitive tool to assess the severity and progression of neural deterioration [44]. Since the most sensitive effect of MB exposure is neurotoxicity, we assumed that EEG could be utilized as a sensitive biomarker for MB exposure.

In this study, the workers’ EEG was measured before and after fumigation work to determine whether occupational exposure to MB affects the health of the workers. In addition, the bromide ion in urine, a traditional biomarker, was analysed to examine whether the workers were exposed to MB and to quantify the exposure.

## Materials and methods

### Subjects

The study group was engaged in controlling pests with MB, and all companies were registered with the Animal and Plant Quarantine Agency (APQA) [45]. The control group was the quarantine inspectors overseeing the MB work, who are all public officials in the Yeongnam Regional Office (located in Busan) of the Animal Plant Quarantine Agency.

From February to August 2019, 44 individuals of the study group and 20 individuals of the control group were recruited in Busan, Korea at the Environmental Health Center of Dong-A University. This observational study was conducted as part of the plant quarantine technology development programme of APQA. Written informed consent was obtained from each subject prior to study participation. The Institutional Review Board approved the study protocol (Dong-a University Institutional Review Board, IRB number: 2-1040709-AB-N-01-201806-BR-004-04). After obtaining basic demographic information, the participants were examined for EEG before and after work by registered clinical research nurses. The levels of bromine ions in participants’ urine were also analysed before and after work.

The fumigation workers conducted tasks including sealing the containers, measuring the MB concentrations, applying the MB and exhausting the MB, and the commodities included imported fruits in a warehouse or wood in lumber yards. Quarantine inspectors examined the imported or exported plants for pests while supervising the MB fumigation work in the same working area as the fumigators, which could make inspectors work in the similar physical working conditions as the fumigators. The inspectors were less likely to be exposed to MB, only overseeing the fumigant application and checking the gas concentrations during the fruit or wood fumigation. Work and EEG measurement processes of the subjects are shown as Table 1 in general.

**Table 1:** Work and EEG measurement processes of the subjects in general.

Time	Fumigator	Inspector
08:00	Measure EEG indices after stabilization	-
08:30	-	Measure EEG indices after stabilization
09:00	Move to working area	Prepare documents and travel to work area
09:30	Prepare MB injections	Inspect plants
	Calculate MB dose Seal container Connect hose	Check documents and plants Open packing materials Visually inspect plants by cutting or sifting
11:00	Inject MB	Oversee MB injections
	Check MB leakage	
11:30	Completion of MB injections	Complete overseeing MB injections
12:00	Lunch	Lunch
13:30	Degas MB	Confirm MB concentrations(~13:40)
		Inspect plants (13:40~)
	Measure MB concentration Remove tape on containers Open containers Wait for MB concentration reduction (approximately 2hrs)	Check document and plants Open packing materials Visually inspect plants by cutting or sifting
15:30	Travel back to office	Travel back to office
16:00	Measure EEG indices after stabilization	Document findings
16:30	-	Measure EEG indices after stabilization

Subjects’ urine was collected just before the measurement of EEG indices. EEG indices and urinary bromide ion concentrations were measured in one or two subjects per group on the day of MB fumigation work. All subjects were recruited among 76 fumigators and 33 inspectors registered in Busan port area. Sample size was balanced according to magnitude of both groups.

### Analysis of bromide ions in urine

Urine samples were collected after the subjects were familiarized with the urine collection method to prevent contamination. For the collection of urine, the first extracorporeal urine was not collected, and more than 10 ml of intermediate urine was collected using a dedicated urine cup (Qorpak PLC-03701 Natural Polypropylene Jar with 58–400 White Polypropylene Unlined Cap 120 m) and then stored at 4°C until transported to an institution for testing. Five millilitres of each of the transferred samples were dispensed into dedicated conical tubes (CELLTREAT 229412 Centrifuge Tube, 15 mL, Polypropylene) and stored in a deep freezer at -80°C for urinary bromide ion concentration analysis.

The concentration of bromide ions in urine was analysed with a high-performance liquid chromatography/inductively coupled plasma mass spectrometer (HPLC/ICP-MS, HPLC; Agilent Technologies 1260 series/Agilent Technologies 7700 series, ICP-MS; Agilent Technologies, CA, USA).

### Resting EEG measurement

All subjects underwent EEG recordings in an upright seated position in a resting state and in a simple repetitive auditory pure-tone stimulus with their eyes closed for five and eight minutes respectively. A series of other tests was also conducted. These additional tests were beyond the scope of the present study.

Noninvasive monopolar scalp electrodes recorded electrical brain activity at prefrontal regions (Fp1, Fp2 in International 10/20 electrodes system) with a reference of the right earlobe. The frequency band-pass of the neuroNicle FX2 amplifiers (LAXTHA Inc., Korea) was 3 to 41 Hz, and the input range was +/-590 uV (Input noise<0.8 μVrms); All filters were digital, and IIR Butterworth filters were applied. Band stop: 2^nd^ order with f_1_ = 55 Hz and f_2_ = 65Hz. High-pass filter: 1^st^ order with f_c_ = 2.6 Hz. Low-pass filter: 8^th^ order with f_c_ = 43 Hz [46]. The contact impedances were kept below 10 kΩ each. All data were digitized in continuous recording mode (5 minutes of EEG; 250 Hz sampling rate; 15-bit resolution).

The authors have proved some prefrontal EEG markers can be reliably reflect the common feature of other brain regions with a multi-channels EEG device [38]. EEG indices such as median frequency (MDF) and alpha-to-theta ratio (ATR) were measured of 112 healthy individuals aged 20 to 69 years in the eye-closed resting state. A 5-minute measurement was taken at 8 regions (Fp1, Fp2, F3, F4, T3, T4, O1, O2). It was found that the markers showed high conformity over all brain lobes and high stable reproducibility.

To minimize ocular, muscular, and other types of artifacts, an operator monitored the subject and EEG traces, instructed the subject to remain in an eyes-closed and muscle-relaxed state in a quiet environment and alerted the subject whenever he/she showed signs of behavioral or EEG drowsiness.

We tested for the data contamination due to muscle and eye movement of the (Fp1, Fp2) prefrontal EEG signals as we did not reject any artifact in the signal processing. First, we checked that none of EEG data of the entire subjects was contaminated by large amounts of artifacts. Specifically, none of participants contained more than 10% of epochs exceeding 200 uV in its maximum amplitude; this value was a common exclusion threshold of each epoch due to serious artifacts [47]. When applying stricter voltage threshold, we found still none with 10% of epochs exceeded 100 uV. So, none of the eye-closed resting-state EEG data were rejected for artifacts in this study.

### EEG biomarkers and computation

The frequency-domain (or spectral-domain) features are usually used in the quantitative analysis of EEG rhythms. To transform the EEG signal from the time-domain to the frequency-domain, a Fourier transform of the autocorrelation function was employed to provide the power spectral density. In resting eyes-closed EEG, intrinsic oscillation reflecting an idling cortical state becomes dominant, and the dominant peak frequency is usually located in the 4–13 Hz band. Previous reports have commonly addressed that the dominant oscillatory frequencies that appear in the alpha band during normal aging become lower in cognitively disordered patients [48,49].

The present study focused on the following EEG markers explaining the slowing of the brain rhythms in the resting eyes-closed state: MDF and ATR which were reported to be good classification markers for Alzheimer’s disease and mild cognitive impairment [29,50]. These markers were derived from a frequency-domain analysis of EEG data measured for 5 minutes; the MDF measures the median frequency in the dominant intrinsic oscillatory frequency band of 4–13 Hz of the EEG power spectrum. The ATR measures the power ratio of alpha rhythms (8–13 Hz) to theta rhythms (4–8 Hz).

Concretely, the EEG power spectrum was obtained by fast Fourier transform (FFT) of EEG signal with a rectangular window. The MDF was calculated in two steps. (1) All the spectral power values in the 4–13 Hz frequency domain were summed and divided by two. (2) The frequency was selected at which the cumulative power in the 4–13 Hz first exceeded the value calculated in step (1). The alpha and theta power in the ATR was obtained as following. (1) Alpha power: the spectral power values in the frequency range from 8 to 13 Hz were summed and converted to natural logarithmic scale. (2) Theta power: the spectral power values in the frequency range from 4 to 8 Hz were summed and converted to natural logarithmic scale. The power data were logarithmically transformed in order to fulfill the normal distributional assumptions required for parametric statistical analysis. (3) ATR: divide alpha power into theta power.

### Statistical analysis

The differences in participants’ baseline characteristics were investigated with the independent *t*-test or the chi-squared test for continuous and categorical variables, respectively. An independent *t*-test was conducted to determine the difference between the study and control group for the EEG and bromide ion before fumigation work as well as after the work. A paired *t*-test was performed to determine whether the EEG and bromide ions of the study and control groups were different before and after fumigation work. EEG and urinary bromide ion concentrations are presented as the mean ± SD of each group. As a result of conducting Kolmogorov-Smirnov test to check the normal distribution on indices, at least one group of both groups in all indices satisfied normality before work (*P*>0.05 for all indices). The relationship between urinary bromide ion concentration and EEG indices was analysed with Pearson's correlations for all subjects. A partial correlation was analysed to examine the relationship between the urinary bromide ion concentration and the EEG indices while controlling for age and gender. Statistical analysis for the data was performed with SPSS (SPSS ver. 23).

## Results

### Demographic information

The participants’ demographic features and baseline values are summarized in Table 2. There were significant differences in sex and gas mask use on the day of the test, while there were no significant differences in other factors, such as age, duration of work, smoking status, and alcohol consumption.

**Table 2:** Demographic information.

Demographic variable	Fumigator	Inspector	*P*-value
Gender: Male	44(100%)	13(65.0%)	0.000
Age (years)	42.54±10.19	37.15±10.81	0.059
Alcohol: Yes	42(95.5%)	18(90.0%)	0.403
Duration of work (years)	10.15±9.62	6.65±8.06	0.161
Gas mask use on test day:Yes	44(100%)	13(65.0%)	0.000
Smoking			
Never	14(31.8%)	13(65.0%)	
Previously	11(25.0%)	2(10.0%)	
Currently	19(43.2%)	5(25.0%)	
Fumigation site on the test day^1^			
Warehouse	0(0%)	0(0%)	
Container	42(87.5%)	20(100%)	
Tent	6(12.5%)	0(0%)	
Gas mask use on a typical day			
Not worn	0	2(10.0%)	
Rarely worn	1(2.3%)	3(15.0%)	
Worn moderately often	9(20.5%)	7(35.0%)	
Almost always worn	18(40.9%)	4(20.0%)	
Always worn	16(36.4%)	4(20.0%)	

Data are summarized as the means ± SD for continuous variables and as the frequencies and proportions for categorical variables. The *P*-values were derived from an independent *t*-test for continuous variables or a chi-squared test for categorical variables.

^1^Multiple responses were possible.

### Urinary bromide ion in fumigators and inspectors before and after MB work

The concentrations of urinary bromide ion in fumigators and inspectors before and after the work are shown in Table 3. The mean concentrations of fumigators and inspectors before work were significantly different between the two groups by 7.390 and 3.984 μg/mg CRE, respectively. The mean concentrations after work also showed a significant difference between the two groups (18.311 and 4.210 μg/mg CRE). Interestingly, the concentration in fumigators after work significantly increased (*P*<0.001). However, the inspectors showed no difference in urinary bromide ion concentrations between before and after work levels.

**Table 3:** Urinary bromide ion concentrations (μg/mg CRE) of fumigators and inspectors before and after fumigation work.

Group	Before	After	*T*	*P*^1^
Fumigator(n = 44)	7.390 ± 6.468	18.311 ± 16.004	-5.472	<0.001
Inspector(n = 20)	3.984 ± 2.831	4.210 ± 3.644	-0.296	0.770
t	2.930	5.537	-	-
*P*^2^	0.005	<0.001	-	-

Urinary bromide ion concentrations are expressed as mean ± SD.

^1^ *P*-values were indicated based on paired t-test.

^2^ *P*-values were indicated based on independent t-test.

### Comparison of EEG indices of fumigators and inspectors before MB work

Before MB work started, we conducted an independent t-test on the fumigator and the inspector EEG indices obtained. As a result, as shown in Table 4, there was no statistically significant difference between their indices.

**Table 4:** EEG indices of fumigators and inspectors before MB work.

EEG indices	Fumigator(*n* = 44) M ± SD [min, max]	Inspector(*n* = 20) M ± SD [min, max]	*t*	*P*
MDF(Hz)	9.515± 0.358 [8.805, 10.325]	9.528± 0.537 [8.706, 10.536]	-0.099	0.922
ATR	1.344± 0.243 [1.010, 2.179]	1.410± 0.321 [1.065, 2.171]	-0.906	0.368

EEG indices are expressed as mean ± SD. MDF was expressed as the median frequency (Hz) in the dominant intrinsic oscillatory frequency band of 4–13 Hz of the EEG power spectrum and ATR was expressed as the power ratio of alpha rhythms (8–13 Hz) to theta rhythms (4–8 Hz).

### Comparison of EEG indices before and after MB work

**Fig 1:**
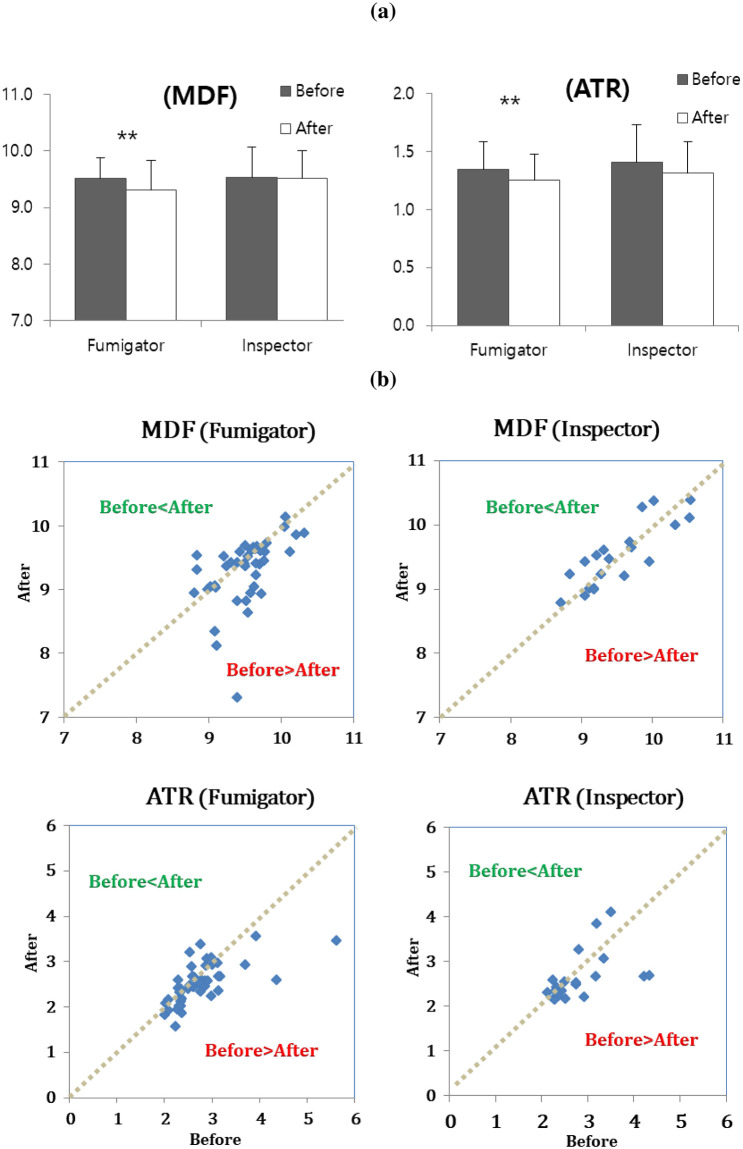
Illustration before and after MB work on subjects’ EEG indices. MDF was expressed as the median frequency (Hz) in the dominant intrinsic oscillatory frequency band of 4–13 Hz of the EEG power spectrum and ATR was expressed as the power ratio of alpha rhythms (8–13 Hz) to theta rhythms (4–8 Hz). (a) Bar-graph. The number of stars beside the p-value represents the level of statistical significance; * *P* <0.05, ** *P* <0.01 and *** *P* <0.001. (b) Scattergram. The dots above the 1: 1 dotted line indicates subjects’ EEG values after MB work is higher than before and the dots below the line means the values after work is lower than before.

**Fig 2:**
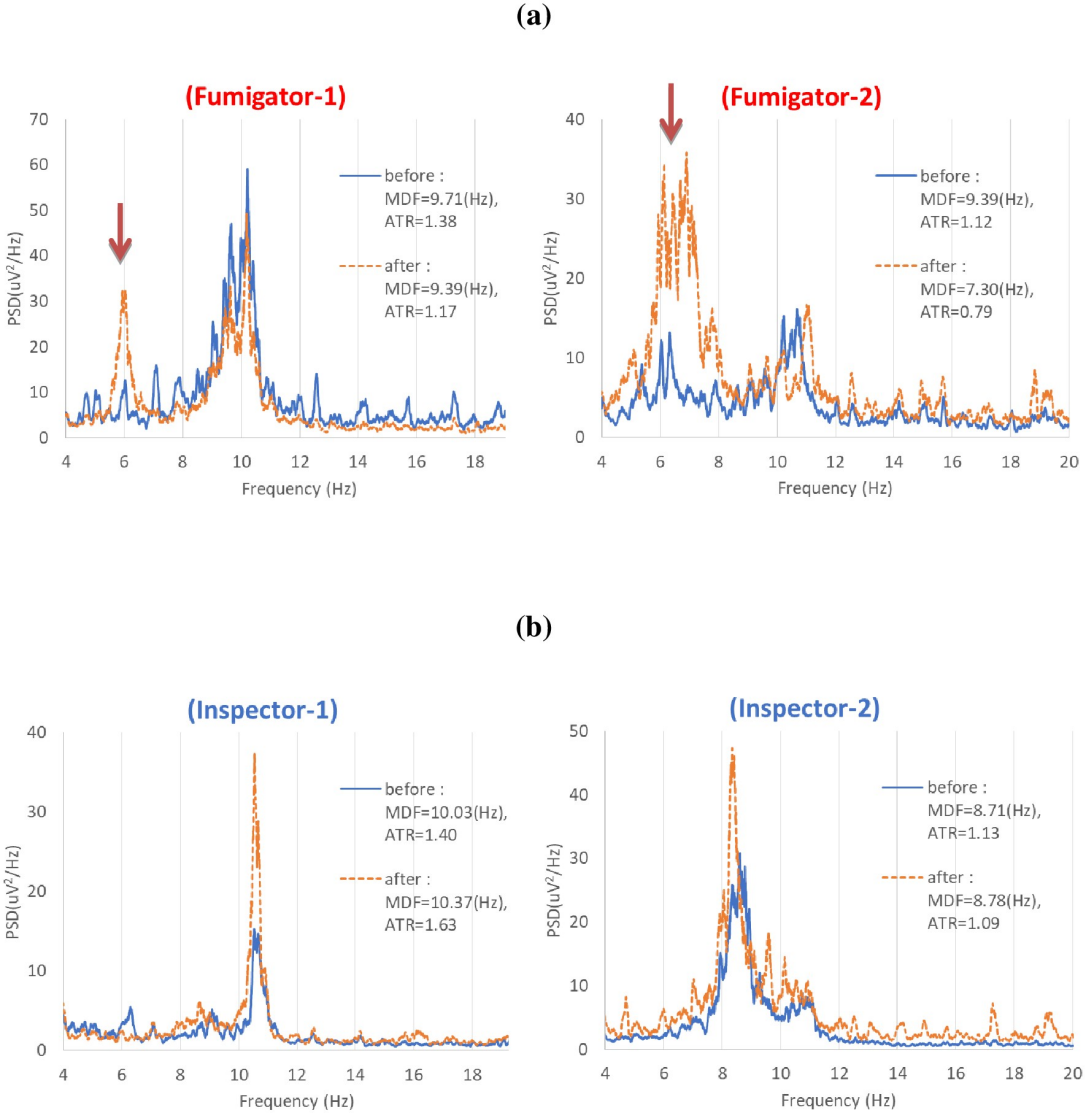
Power spectral density (PSD) calculated from the eye-closed resting EEG measured before and after the work for two fumigators (a) and two inspectors (b). The fumigator’s PSD showed more slowed intrinsic EEG oscillation and the lower MDF and ATR after the methyl bromide fumigation work due to increase of theta-activity together with sharp waves (see arrow). It was similar to the result in the former study [51]. But the inspector’s PSD did not shift to lower frequency.

We examined the difference between EEG indices of fumigators and inspectors before and after MB work, as shown in Table 5. The fumigator EEG indices of MDF, ATR significantly decreased after work compared to those before work. However, inspectors did not show a difference between before and after work levels. Fig 1 illustrates that the EEG indices of fumigators showed significant differences before and after work, while this was not observed in inspectors. Fig 2 shows power spectral density (PSD) calculated from the eye-closed resting EEG measured before and after the work for two fumigators (a) and two inspectors (b). The fumigator’s PSD showed increase of theta-activity together with sharp waves (see arrow). In contrast, no abnormal EEG signs were found in inspectors.

**Table 5:** Comparison before and after MB work on subjects’ EEG indices.

EEG indices		Fumigator(*n* = 44)				Inspector(*n* = 20)		
	Before M±SD	After M±SD	*t*	*P*	Before M±SD	After M±SD	*t*	*P*
MDF(Hz)	9.515± 0.358	9.306 ± 0.518	3.018	0.004	9.528± 0.537	9.515 ± 0.497	0.201	0.843
ATR	1.344± 0.243	1.256 ± 0.224	2.808	0.007	1.410± 0.321	1.317 ± 0.272	1.383	0.183

EEG indices are expressed as mean ± SD. *P*-values were indicated based on paired *t*-test. MDF was expressed as the median frequency (Hz) in the dominant intrinsic oscillatory frequency band of 4–13 Hz of the EEG power spectrum and ATR was expressed as the power ratio of alpha rhythms (8–13 Hz) to theta rhythms (4–8 Hz).

### Correlation between bromide ion levels and EEG indices in all subjects

**Fig 3:**
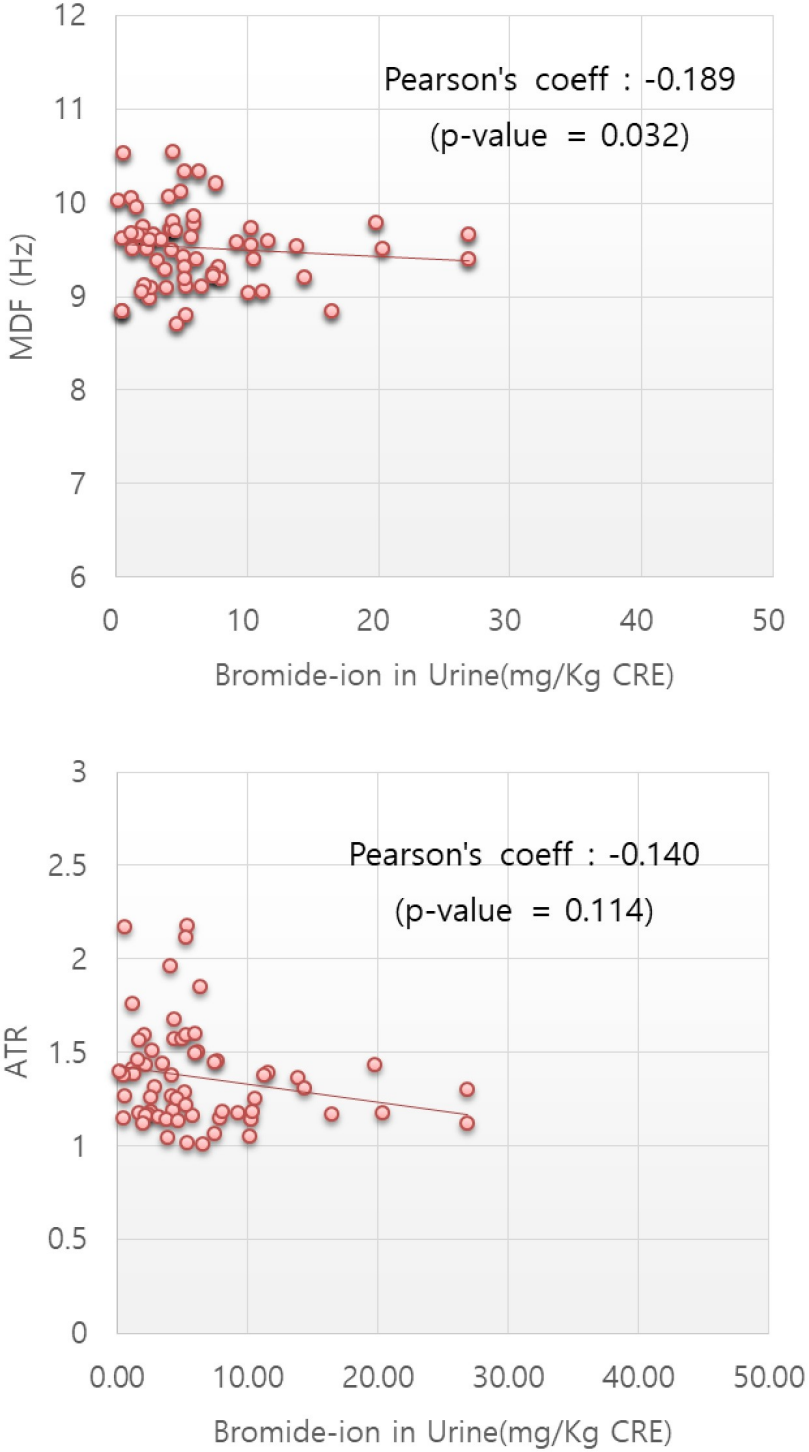
Spearman’s correlation between urinary bromide ion levels and EEG indices. Urinary bromide ion levels and MDF values showed a negative linear correlation with *P*-value <0.05. ATR values also showed a negative linear correlation with urinary Br- levels but the correlation was not significant. The number of subjects was 64.

We assessed the relationship between the urinary bromide ion levels and the EEG indices in all the subjects (*n* = 64) before and after work. There was no need to differentiate between the two groups in this assessment. Moreover, since inspectors’ bromide ion concentrations were limited to a low range, the correlation was analysed among all subjects to investigate a wide range of concentrations. Urinary bromide ion levels and MDF values showed a negative linear correlation with *P*-value <0.05. The results of the partial correlation analysis when controlling for age and gender also showed the same tendency as the above, even if *P*-values under the condition controlled age and gender were higher those under the uncontrolled condition. ATR values also showed a negative linear correlation with urinary Br- levels but the correlation was not significant. The details regarding the correlation analysis are shown in Table 6 and Fig 3.

**Table 6:** Correlations of all subjects’ urinary Br^-^ levels (μg/mg CRE) and EEG indices before work as controlled for age and gender.

Control variables		MDF(Hz)	ATR
**None**^1^	Correlation	**-0.189**	-0.140
	P-value	**0.032**	0.114
	d.f.	128	128
**Age and gender**	Correlation	**-0.181**	-0.156
	P-value	**0.042**	0.081
	d.f.	124	124

^1^ Cells contain zero-order (Pearson) correlations. The numbers of subjects were 64.

## Discussion

The bromide ion concentration in blood or serum has conventionally been used as a biomarker of MB exposure [20,52]. Urinary bromide ion concentrations have also been analysed as an indicator in various studies [19–21,53]. Urinary bromide ion concentrations were frequently analysed by gas chromatography [21,53], ion chromatography [20,53] or ICP-MS [54,55]. The HPLC/ICP-MS method was applied by modifying ICP-MS in this study.

Bromide ion concentrations in biological samples from MB fumigation workers were analysed in some previous studies. Lee et al. [20] reported the mean urinary concentrations in 11 exposed workers (35.56 mg/L) and 21 non-exposure workers (4.74 mg/L) with significant differences (*P*<0.001) [20]. Koga et al. [53] also stated that levels in 36 exposed workers were 13.3 mg/L, while those in 6 non-exposed workers were 7.1 mg/L [20]. Tanaka et al. (1991) indicated that the urinary concentration of 251 fumigation workers was 9.1 mg/L and that of 379 non-MB workers was 6.3 mg/L [19]. Yamano et al. [21] stated that the Br^-^ concentrations, which were normalized by urinary creatinine, in the synthesis group of MB manufacturing facility, had median value of 13.0 μg/mg CRE and those of the filling group had a concentration of 11.9 μg/mg CRE. Their levels were higher than 7.2 μg/mg CRE noted in the other group [21]. These findings were similar to the values of 18.3 μg/mg CRE in the 44 fumigators and 4.2 μg/mg CRE in the 20 quarantine inspectors, which comprised the control group, after MB work in this study.

These previous studies have not considered the MB concentrations accumulated based on the MB work performed on the measurement day, which needs to be further studied. In this study, we investigated how much MB was accumulated in the human body due to the fumigation work performed during one day rather than the baseline urinary bromide ion levels in workers. This study indicated the urinary bromide ion levels in the study group increased to 18.311 μg/mg CRE from 7.390 μg/mg CRE following one day fumigation work, with a significant difference (*P* <0.05).

With regard to the relationship between poisoning symptoms and bromide concentrations, a lack of a correlation has occasionally been noted; indeed, Yamano et al. [21] examined MB manufacturing facility workers and stated that even if the bromide ion concentrations in the synthesis group had a median value of 13.0 μg/mg CRE, levels higher than the levels noted in the other group, most of the workers did not show any symptoms by MB exposure [21]. He reported that only 3 workers among 124 experienced subjective symptoms. One of them measured only 11.5 μg/mg CRE. Tanaka et al. (1991) did not report symptoms of toxicity either, although 44.6% of 251 workers had bromide ion levels that exceeded 10 mg/L, the 95% confidence limit of the mean of non-MB workers’ bromide ion levels [19]. Results from animal studies have shown that lethal and survival dosages are similar, and other reports have suggested that symptom onset susceptibility may differ between individuals [56]; for example, Kato et al. [57] found that no rats exposed to 700 ppm MB for 4 hours were killed, while 100% of animals exposed to 800 ppm MB were killed [57]. Irish (1940) also found that 100% of rats exposed to 100 ppm MB for 24 hours survived, but 100% lethality was observed at 220 ppm MB [58]. The margin of safety for MB may be narrow, and poisoning can occur even with minor differences in exposure. The fumigators in this study did not show any symptoms, which seems to be due to this characteristic of MB. However, it cannot be stated that MB is harmless to the human body.

The intrinsic rhythms of resting EEG reflect mainly brain idling and inhibitory processes as an important neural substrate for human cognition [59–63]. Previous studies have shown that the slowing of resting intrinsic rhythms is related to declines in cognitive functions such as attention, memory and performance [42,64–70]. In particular, abnormal EEG was found in 11 of the 33 MB users engaged in soil disinfection inside greenhouse [51]. In this study, the fumigator’s PSD showed more slowed intrinsic EEG oscillation and the lower MDF and ATR after the methyl bromide fumigation work due to increase of theta-activity together with sharp waves, which was similar to the result in the former study (Fig 2). The mean EEG indices after fumigation work on the study group fell compared to the indices from before fumigation (Fig 1a). When the magnitude of decrease in MDF (9.52 → 9.31 Hz) and ATR (1.34 → 1.26) were compared to the difference depending on the reference range by aging and cognitive decline (Table 5), their decrease implies that fumigators were functionally aged from the 20s (9.58Hz) to 40s (9.34Hz), or declined cognitively from M4 (1.38) to M3 (1.28) among MMSE score ranges, a global cognitive decline parameter (S2 Fig in S1 File). The reference ranges were derived from raw data in the papers the authors reported [36,38]. Across all the subjects, there was a negative correlation between the urinary bromide ion concentrations and MDF values (P<0.05) (Fig 3). Thus, MDF could be useful for assessing functional degradation or weakness of autonomic nerves as well as for early prediction of disease risk by MB fumigation work.

Several studies reported ambient MB concentrations at various fumigation sites determined with a personal sampling device or collected with a Tedlar bag. Some of these studies compared urinary bromide concentrations to airborne MB concentration. Lee & Shin [71] reported that 2 of 27 workers (7.5%) exceeded the Korea Ministry of Labour standard (5 ppm), and 4 workers (14.8%) exceeded the US ACGIF standard (1 ppm) [71], resulting in TWA being collected and analysed by individual sampling apparatuses during tent fumigations. Tanaka et al. (1991) presented the MB concentration collected by a personal sampling device according to the fumigation site (log yard, ship, warehouse) and type of work (dispersion, degassing) [19]. Regardless of the place of fumigation, MB concentrations were higher on the degassing sites than on dispersion sites. Concentrations in the area at log yards, ships and warehouses were measured at 74.6, 31.8 and 19.8 ppm. There was a positive correlation between the urinary bromide ion concentration and the MB concentration in the air (*r* = 0.596, *P*<0.01). Yamano et al. [21] analysed the concentration of MB collected in a Tedlar bag and measured urinary bromide ion levels according to the workplace process (synthesis, filling, and other work). The urinary bromide concentration in the workers was 22.6 ~ 83.4 μg/mg CRE when the MB concentration in air was quite high (25.5 ppm), which was higher than the usual level (urine: 10.1 ± 4.8 μg/mg CRE) [21]. This study did not provide MB concentrations during the fumigations. However, workers were likely considered to be exposed to high MB concentrations, based on the previous studies and the urinary bromide ion concentrations in this study; the levels in the workers was higher than that in the inspectors’ and increased to 18.311 μg/mg CRE from 7.390 μg/mg CRE after work.

Most of the fumigators indicated that they wore gas masks, as shown in Table 1, but their health was still affected. There are several factors that may have resulted in MB exposure. According to the phytosanitary treatment regulations in Korea, the fumigators are always required to wear gas masks during fumigation work. Quarantine inspectors oversee the fumigation on-site, but this oversight is limited to the time of MB application or just before the gas is released. Fumigation workers involved in other processes practice self-regulation regarding wearing a gas mask. Workers may not wear gas masks because of inconveniences, such as having a hampered view or difficulty breathing. Another cause of exposure to MB was indicated by a previous study [19]. The gas mask can leak from the contact area on the face. Inadequacy of the respirator canisters used in fumigation work increases the danger of exposure as well. There are two types of canisters attached to the gas mask: MB and OV. The breakthrough time of the OV canister is shorter than that of the MB canister, and MB absorbed into the OV canister can be desorbed during respiration. All fumigation companies in Korea use the canister for OV, which can increase the potential risk that fumigation workers may be exposed to higher concentrations of MB.

Developing MB alternatives could bypass MB exposure against fumigators. For example, ethyl formate has been studied for efficacy against mealybugs on fresh fruits such as orange and stored grain insects [72–76]. And the toxicity of sulfuryl fluoride and ethane dinitrile against wood pests and liquefied phosphine fumigants against common insects in cut flowers have also been investigated [77–80].

An investigation was conducted on fumigation workers and quarantine inspectors, who would not be considered a control group in a similar environment, such as in a port area in which access is strictly restricted. A significant achievement of this work is the contribution to the understanding of the health impacts on fumigation technicians who do not show symptoms of toxicity even after occupational exposure to MB. However, the insufficient sample size of the study and control group was a limitation because the number of fumigators registered in APQA was only 76 in Busan port area and it was quite difficult to enroll subjects for active measurement. In addition, the fact that the MB concentrations of the workplaces were not provided and the lack of correlation between urinary bromide ion levels in the workers and airborne MB concentrations were noteworthy. Finally, identification of the cause of MB exposure to workers and the health effects of chronic MB exposure should be included in future studies. It should be also revealed that how much past cumulative MB fumigation work affects EEG.

Although this study has limitations, we showed that fumigators who conducted MB fumigation work tended to be less healthy, have higher concentrations of bromide ion and have slower post-work EEG indices than inspectors. It is expected that the MB exposure status of workers and the EEG results reported in this study will be used to protect workers’ health rights, prevent toxicity and manage fumigation safety.

## Conclusion

EEG indices and urinary bromide ion concentrations in the study (fumigators) and control (quarantine inspectors) groups were measured before and after MB fumigation work. Based on the results of this study, we have revealed that the fumigators’ post-work EEG indicators of MDF and ATR were significantly decreased compared to the pre-work values (*P*<0.01 in all indices). We have also confirmed that the mean post-work concentration of bromide ion (18.311 μg/mg CRE) in the fumigators was significantly increased from the pre-work value (7.390 μg/mg CRE) (*P*<0.001). In contrast, there were no significant differences in EEG indices and urinary bromide ion of inspectors between pre- and post-work. The urinary bromide ion levels in all the subjects were negatively correlated with MDF (*P* = 0.032). It is expected that this result will provide a scientific basis for the protection of workers' health rights, prevention of poisoning accidents, and improvement of fumigation safety by conducting additional studies on the health effects of chronic MB exposure and evaluation on environmental exposures of MB.

## Supporting information

S1 File (DOC)
