# Supplementary material for: Negative effect of methyl bromide fumigation work on the central nervous system
Source: PLoS One. 2020 Aug 3;15(8):e0236694. doi: 10.1371/journal.pone.0236694 (PMC7398500; doi:10.1371/journal.pone.0236694)
Supplement: S1 File — (DOC) [file pone.0236694.s001.doc]

**Negative effect of methyl bromide fumigation work on the central nervous system**

**Supplementary materials**

Min-Goo Park^1,2,¶,*^ , Jungmi Choi^3,¶^, Young-Seoub Hong^4,5^, Chung Gyoo Park^2^, Byoung-Gwon Kim^4,5^, Se-Young Lee^5^, Hyoun-Ju Lim^5^, Hyoung-ho Mo^1^, Eunjo Lim^3^, Wonseok Cha^3^

^1^ Department of plant quarantine, Division of pest control, Animal and Plant Quarantine Agency (APQA), Gimcheon-si, Gyeongsangbuk-do, Republic of Korea

^2^ Institute of Life Science (BK21^+^ Program), Gyeongsang National University, Jinju-si, Gyeongsangnam-do, Republic of Korea

^3^Human Anti-Aging Standards Research Institute, Uiryeong-gun, Gyeongsangnam-do, Republic of Korea

^4^Department of Preventive Medicine, College of Medicine, Dong-A University, Busan, Republic of Korea

^5^Heavy Metal Exposure Environmental Health Center, Dong-A University, Busan, Republic of Korea

^¶^These authors contributed equally to this work

^*^ Corresponding author: e-mail: [pmg@korea.kr](mailto:pmg@korea.kr), telephone: +82-(0)51-606-5201

**Table S1. The data on fumigators’ EEG indices and urinary bromide ion**

| **Subject-ID^1^** | **Age** | **Bromide ion(μg/mg CRE)** | | **MDF(Hz)** | | **ATR** | |
| --- | --- | --- | --- | --- | --- | --- | --- |
|  |  | **Before** | **After** | **Before** | **After** | **Before** | **After** |
| Fumi-01-M | 37 | 5.335 | 17.586 | 8.805 | 8.938 | 1.016 | 1.040 |
| Fumi-02-M | 42 | 2.198 | 2.706 | 9.644 | 9.644 | 1.433 | 1.229 |
| Fumi-03-M | 37 | 5.977 | 11.832 | 9.766 | 9.445 | 1.498 | 1.121 |
| Fumi-04-M | 39 | 2.071 | 8.414 | 9.741 | 9.627 | 1.592 | 1.336 |
| Fumi-05-M | 28 | 4.231 | 11.241 | 9.491 | 9.403 | 1.189 | 1.086 |
| Fumi-06-M | 50 | 1.631 | 1.330 | 9.659 | 9.218 | 1.178 | 1.103 |
| Fumi-07-M | 26 | 16.467 | 8.301 | 8.843 | 9.535 | 1.168 | 1.157 |
| Fumi-08-M | 28 | 4.990 | 12.651 | 10.126 | 9.588 | 1.574 | 1.173 |
| Fumi-09-M | 35 | 20.374 | 22.550 | 9.514 | 8.812 | 1.176 | 1.056 |
| Fumi-10-M | 34 | 5.732 | 18.070 | 9.629 | 9.043 | 1.159 | 1.013 |
| Fumi-11-M | 43 | 6.585 | 7.897 | 9.110 | 8.120 | 1.010 | 0.915 |
| Fumi-12-M | 44 | 10.306 | 20.766 | 9.735 | 8.926 | 1.141 | 0.973 |
| Fumi-13-M | 65 | 0.516 | 1.300 | 9.619 | 9.657 | 1.144 | 1.209 |
| Fumi-14-M | 61 | 11.583 | 46.014 | 9.594 | 9.615 | 1.389 | 1.292 |
| Fumi-15-M | 35 | 0.530 | 14.405 | 8.841 | 9.308 | 1.266 | 1.599 |
| Fumi-16-M | 51 | 4.210 | 6.540 | 9.710 | 9.388 | 1.379 | 1.163 |
| Fumi-17-M | 46 | 19.802 | 32.925 | 9.785 | 9.589 | 1.430 | 1.286 |
| Fumi-18-M | 50 | 7.469 | 34.575 | 9.247 | 9.367 | 1.449 | 1.532 |
| Fumi-19-M | 38 | 26.845 | 43.536 | 9.390 | 7.301 | 1.117 | 0.787 |
| Fumi-20-M | 32 | 2.633 | 18.564 | 9.091 | 9.060 | 1.506 | 1.465 |
| Fumi-21-M | 50 | 10.328 | 14.029 | 9.545 | 8.637 | 1.185 | 0.933 |
| Fumi-22-M | 29 | 2.887 | 11.816 | 9.665 | 9.674 | 1.314 | 1.329 |
| Fumi-23-M | 59 | 2.539 | 19.083 | 8.984 | 9.005 | 1.179 | 1.194 |
| Fumi-24-M | 44 | 3.480 | 10.756 | 9.604 | 9.636 | 1.436 | 1.453 |
| Fumi-25-M | 49 | 26.875 | 66.485 | 9.659 | 9.409 | 1.300 | 1.331 |
| Fumi-26-M | 66 | 1.240 | 5.056 | 9.503 | 9.693 | 1.386 | 1.691 |
| Fumi-27-M | 59 | 5.333 | 9.549 | 9.102 | 9.033 | 2.179 | 1.297 |
| Fumi-28-M | 45 | 13.829 | 16.005 | 9.541 | 9.522 | 1.360 | 1.267 |
| Fumi-29-M | 37 | 14.397 | 63.167 | 9.205 | 9.518 | 1.304 | 1.220 |
| Fumi-30-M | 39 | 3.900 | 7.098 | 9.091 | 8.339 | 1.040 | 0.960 |
| Fumi-31-M | 41 | 4.019 | 13.990 | 10.060 | 10.136 | 1.961 | 1.778 |
| Fumi-32-M | 56 | 7.866 | 35.851 | 9.308 | 9.419 | 1.148 | 1.296 |
| Fumi-33-M | 39 | 2.369 | 18.465 | 9.508 | 9.360 | 1.170 | 1.171 |
| Fumi-34-M | 39 | 7.646 | 4.950 | 10.208 | 9.861 | 1.457 | 1.291 |
| Fumi-35-M | 46 | 4.386 | 7.730 | 9.804 | 9.732 | 1.575 | 1.334 |
| Fumi-36-M | 31 | 10.119 | 15.408 | 9.031 | 9.036 | 1.048 | 1.079 |
| Fumi-37-M | 56 | 1.158 | 12.766 | 10.054 | 9.983 | 1.409 | 1.727 |
| Fumi-38-M | 47 | 9.259 | 19.176 | 9.575 | 8.949 | 1.177 | 1.014 |
| Fumi-39-M | 39 | 1.669 | 55.829 | 9.644 | 9.627 | 1.562 | 1.485 |
| Fumi-40-M | 30 | 4.165 | 10.799 | 9.712 | 9.605 | 1.267 | 1.233 |
| Fumi-41-M | 34 | 10.549 | 31.859 | 9.394 | 8.825 | 1.250 | 1.201 |
| Fumi-42-M | 44 | 6.341 | 1.675 | 10.325 | 9.886 | 1.848 | 1.464 |
| Fumi-43-M | 32 | 5.183 | 4.915 | 9.428 | 9.583 | 1.285 | 1.441 |
| Fumi-44-M | 40 | 6.140 | 8.015 | 9.392 | 9.419 | 1.500 | 1.542 |

MDF was expressed as the median frequency (Hz) in the dominant intrinsic oscillatory frequency band of 4-13 Hz of the EEG power spectrum and ATR was expressed as the power ratio of alpha rhythms (8-13 Hz) to theta rhythms (4-8 Hz). ^a^M(Male), F(Female)

**Table S2. The data on inspectors’ EEG indices and urinary bromide ion**

| **Subject-ID^1^** | **Age** | **Bromide ion(μg/mg CRE)** | | **MDF(Hz)** | | **ATR** | |
| --- | --- | --- | --- | --- | --- | --- | --- |
|  |  | **Before** | **after** | **Before** | **After** | **Before** | **After** |
| Insp-01-M | 58 | 0.512 | 2.559 | 8.843 | 9.228 | 1.375 | 1.243 |
| Insp-02-M | 46 | 5.256 | 4.699 | 9.316 | 9.602 | 2.117 | 1.328 |
| Insp-03-M | 53 | 2.603 | 2.466 | 9.602 | 9.199 | 1.259 | 1.078 |
| Insp-04-M | 49 | 5.224 | 2.249 | 9.182 | 9.005 | 1.218 | 1.168 |
| Insp-05-F | 29 | 2.210 | 2.934 | 9.121 | 9.013 | 1.162 | 1.211 |
| Insp-06-M | 29 | 5.253 | 8.824 | 10.326 | 9.997 | 1.592 | 1.334 |
| Insp-07-M | 47 | 3.157 | 4.198 | 9.388 | 9.474 | 1.153 | 1.171 |
| Insp-08-M | 45 | 1.191 | 7.410 | 9.680 | 9.737 | 1.757 | 2.051 |
| Insp-09-M | 21 | 4.342 | 14.219 | 10.536 | 10.380 | 1.678 | 1.533 |
| Insp-10-M | 35 | 4.523 | 6.353 | 9.709 | 9.644 | 1.250 | 1.270 |
| Insp-11-F | 36 | 7.478 | 4.425 | 9.218 | 9.520 | 1.065 | 1.153 |
| Insp-12-M | 28 | 8.030 | 4.213 | 9.182 | 8.997 | 1.180 | 1.112 |
| Insp-13-M | 35 | 1.926 | 0.384 | 9.049 | 9.421 | 1.118 | 1.288 |
| Insp-14-M | 20 | 5.923 | 6.290 | 9.861 | 10.277 | 1.597 | 1.919 |
| Insp-15-F | 45 | 1.563 | 1.320 | 9.959 | 9.430 | 1.459 | 1.102 |
| Insp-16-M | 47 | 11.248 | 9.228 | 9.054 | 8.896 | 1.379 | 1.259 |
| Insp-17-F | 33 | 0.210 | 0.410 | 10.029 | 10.372 | 1.400 | 1.631 |
| Insp-18-F | 30 | 4.636 | 0.784 | 8.706 | 8.782 | 1.132 | 1.085 |
| Insp-19-F | 29 | 3.803 | 0.581 | 9.285 | 9.232 | 1.144 | 1.071 |
| Insp-20-F | 28 | 0.586 | 0.659 | 10.523 | 10.102 | 2.171 | 1.339 |

MDF was expressed as the median frequency (Hz) in the dominant intrinsic oscillatory frequency band of 4-13 Hz of the EEG power spectrum and ATR was expressed as the power ratio of alpha rhythms (8-13 Hz) to theta rhythms (4-8 Hz). MDF and ATR were also expressed as means of values measured left and right prefrontal lobe. ^a^M(Male), F(Female)

**Table S3. Comparison before and after MB work on subjects’ urinary bromide ion concentrations and EEG indices in pilot test**

| indices |  | Fumigator(n=7) | | | |  | Inspector(n=6) | | | |
| --- | --- | --- | --- | --- | --- | --- | --- | --- | --- | --- |
|  | Before  M±SD | | After  M±SD | *t* | *P* | Before  M±SD | | After  M±SD | *t* | *P* |
| MDF(Hz) | 9.540± 0.365 | | 9.150 ± 0.667 | 2.110 | 0.079 | 9.627± 0.684 | | 9.363 ± 0.113 | 0.967 | 0.378 |
| ATR | 1.449± 0.268 | | 1.350 ± 0.384 | 0.650 | 0.540 | 1.451± 0.335 | | 1.413 ± 0.377 | 0.179 | 0.865 |
| Bromide ion(mg/l) | 8.911± 8.160 | | 9.930± 8.961 | -0.840 | 0.433 | 3.265± 2.506 | | 2.813± 1.726 | 0.835 | 0.442 |

Indices are expressed as mean ± SD. *P*-values were indicated based on paired *t*-test.

**Figure S1. Cumulative frequency distribution on pre- and post-work urinary bromide ion of both groups.** The number of fumigators and inspectors were 44 and 20.


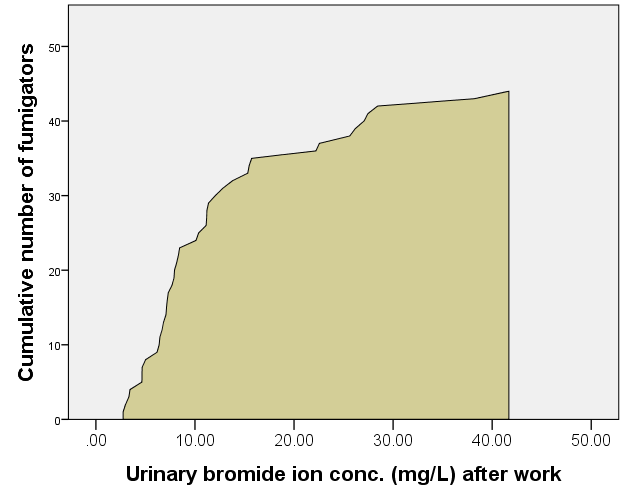


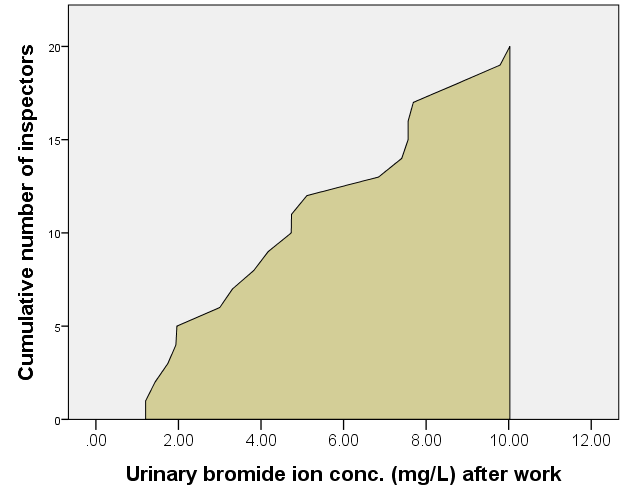

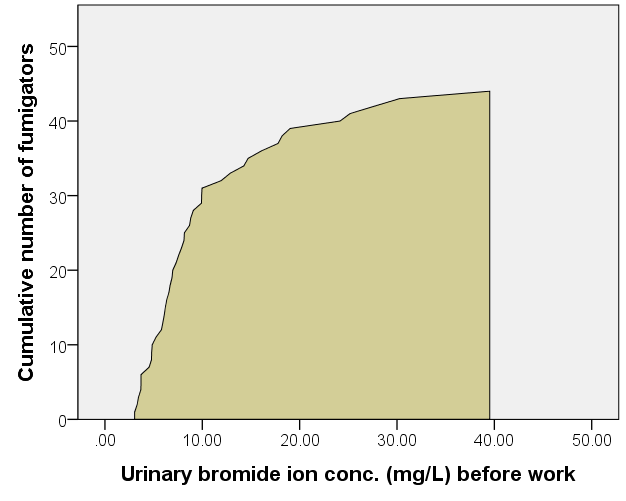


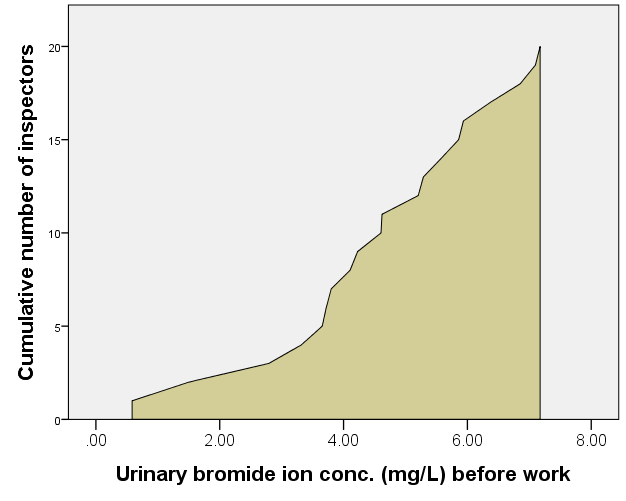


**Figure S2. MDF and ATR values decrease gradually with age and cognitive decline in healthy Korean groups.** The graphs were illustrated, based on raw data in the papers the authors reported [1,2]. MDF measurement was taken of 112 healthy individuals aged 20 to 69 year, ATR of 496 participants aged 50 years or more. They were evaluated with the same instruments and protocols as those used in this study.

**(a)**


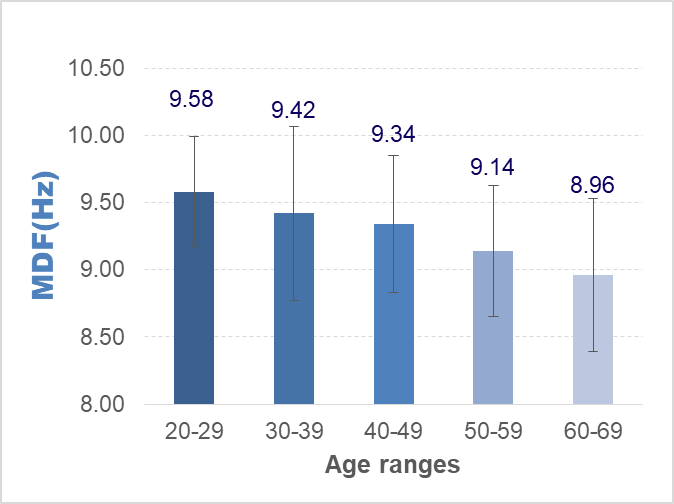


**(b)**


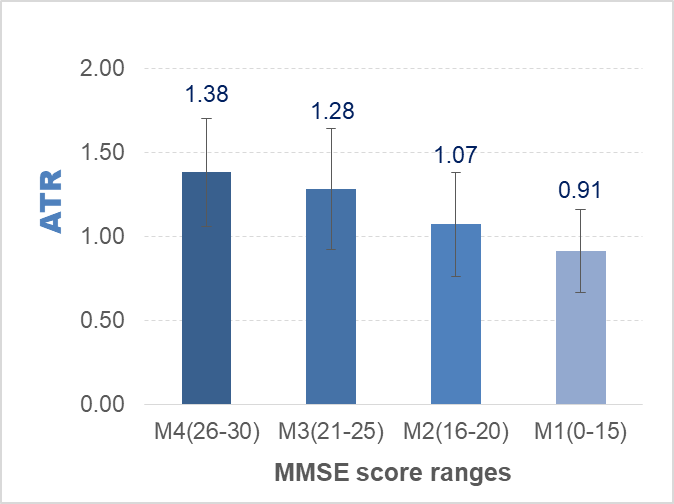


**References**

1. Choi J, Lim E, Park M-G, Cha W. Assessing the Retest Reliability of Prefrontal EEG Markers of Brain Rhythm Slowing in the Eyes-Closed Resting State. Clin EEG Neurosci. 2020; 155005942091483. doi:10.1177/1550059420914832

2. Choi J, Ku B, You YG, Jo M, Kwon M, Choi Y, et al. Resting-state prefrontal EEG biomarkers in correlation with MMSE scores in elderly individuals. Sci Rep. 2019;9: 1–15. doi:10.1038/s41598-019-46789-2
